# Polymeric Nanocarriers for the Delivery of Antimalarials

**DOI:** 10.3390/molecules23102527

**Published:** 2018-10-02

**Authors:** Zandile Mhlwatika, Blessing Atim Aderibigbe

**Affiliations:** Department of Chemistry, University of Fort Hare, Alice Campus, Eastern Cape 5700, South Africa; 201103519@ufh.ac.za

**Keywords:** biodegradable polymers, antimalarials, drug resistance, drug delivery

## Abstract

Malaria is an infectious disease caused by a protozoan parasite which is transmitted by female *Anopheles* mosquitoes around tropical and sub-tropical regions. Half of the world’s population is at risk of being infected by malaria. This mainly includes children, pregnant women and people living with chronic diseases. The main factor that has contributed to the spread of this disease is the increase in the number of drug-resistant parasites. To overcome drug resistance, researchers have developed drug delivery systems from biodegradable polymers for the loading of antimalarials. The drug delivery systems were characterized by distinct features such as good biocompatibility, high percentage drug encapsulation, reduced drug toxicity and targeted drug delivery. In this review article, we highlight the various types of drug delivery systems developed from polymeric nanocarriers used for the delivery of antimalarials.

## 1. Introduction

Malaria is caused by female Anopheles mosquitoes of protozoan genus parasites [1]. Malaria has attracted significant attention, especially in developing countries. This is because it is the cause of numerous deaths in children, pregnant women and patients with HIV/AIDS [2]. Approximately half of the world’s population is at risk of being infected by malaria [2]. In 2015, there were 212 million reported cases of malaria and 430,000 malaria deaths worldwide [3]. Over 90% of those deaths occurred in Sub-Saharan Africa. However, the World Health Organization (WHO) reported that in 2015, the number of malaria infections had reduced to 214 million. This number had fallen from 263 million infections in the year 2000, indicating an 18% reduction [3]. WHO has played a major role in the reduction of incidence of malaria. This has been achieved by scaling up malaria prevention methods, via greater access to diagnostic testing and treatment, increased use of insecticide-treated bed nets and indoor residual spraying in malaria endemic regions. There are four major parasite species that cause human malaria: *Plasmodium falciparum*, *Plasmodium vivax*, *Plasmodium ovale* and *Plasmodium malariae* [4]. These parasites pose serious illness, but *P. falciparum* is the most life-threatening of them all. There have been several controls and prevention approaches taken to manage this disease, such as the use of antimalarials. However, the currently used antimalarial drugs have not been found to be effective due to their toxicity, cost and drug resistance. As a result, these factors have resulted in malaria treatment failure [5]. Other factors that contribute to malaria treatment failure include misdiagnosis, poor patient compliance, poor drug quality and incorrect dosing [5,6]. Due to the aforementioned factors, there is an urgent need to develop drug delivery systems that will be able to reduce the toxicity of the drugs, improve patient compliance and hopefully overcome drug resistance, which is common to the currently used antimalarials [7]. This review article will report the different types of biodegradable nanocarriers developed for the delivery of antimalarials with enhanced therapeutic efficacy.

## 2. The Life Cycle of Malaria Parasite

**Figure 1:**
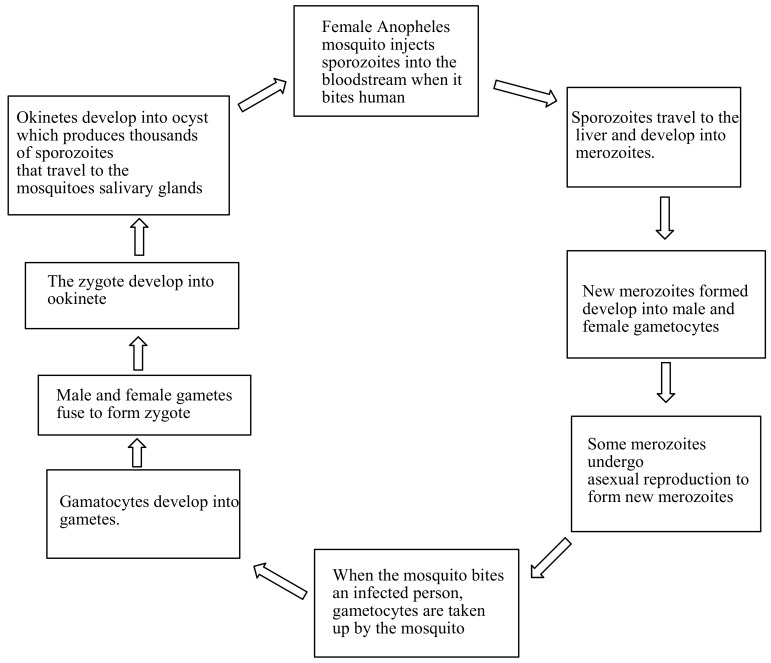
The life cycle of malaria parasite.

The infection begins with a bite from an infected mosquito, injecting *Plasmodium* parasite into the bloodstream, in the form of sporozoites [8]. The sporozoites are carried to the liver cells and multiply asexually to form a thousand merozoites over 7–10 days. This occurs without the appearance of the symptoms [9]. The merozoites are carried to the bloodstream, and invade the red blood cells and multiply again until the cells burst. This cycle is repeated, which results in symptoms such as fever, chills and headaches occuring. Some of the merozoites undergo asexual reproduction to form new merozoites [9]. The new merozoites develop into male and female gametocytes, which then spread into the bloodstream [10]. When the female Anopheles mosquito bites an infected person, the gametocytes will be taken up by the mosquito. In the stomach wall of the mosquitoes, the gametocytes form a zygote. After fertilization, the zygotes develop to form ookinete. Ookinete is transformed to oocyst which multiplies on the exterior surface of the mosquito stomach to form sporozoites. Inside the oocyst, sporozoites multiply and burst, thereby releasing thousands of sporozoites that travel to the mosquito’s salivary glands. The cycle of human infection begins once an infected mosquito bites another person [11,12] (Figure 1). 

### 2.1. Antimalarial Resistance

**Figure 2:**
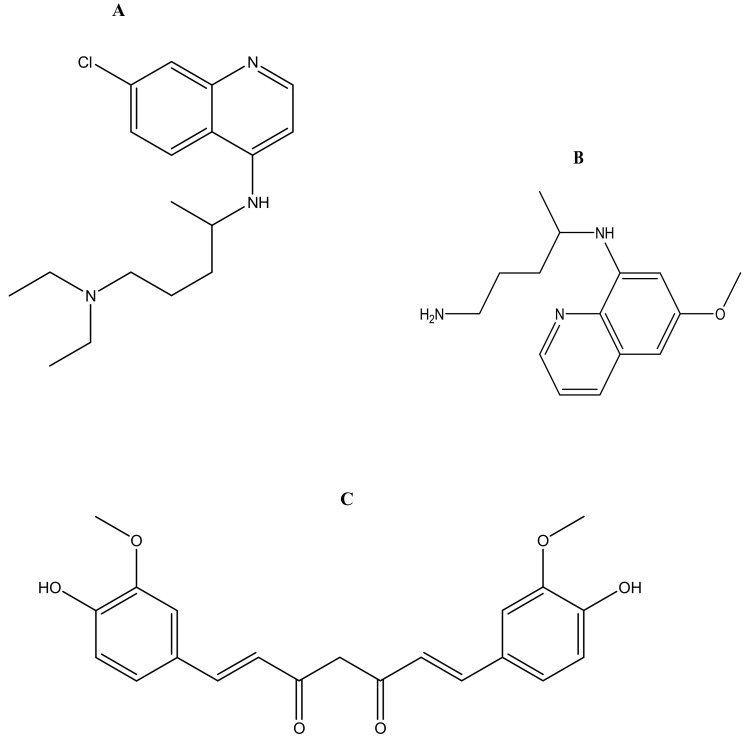

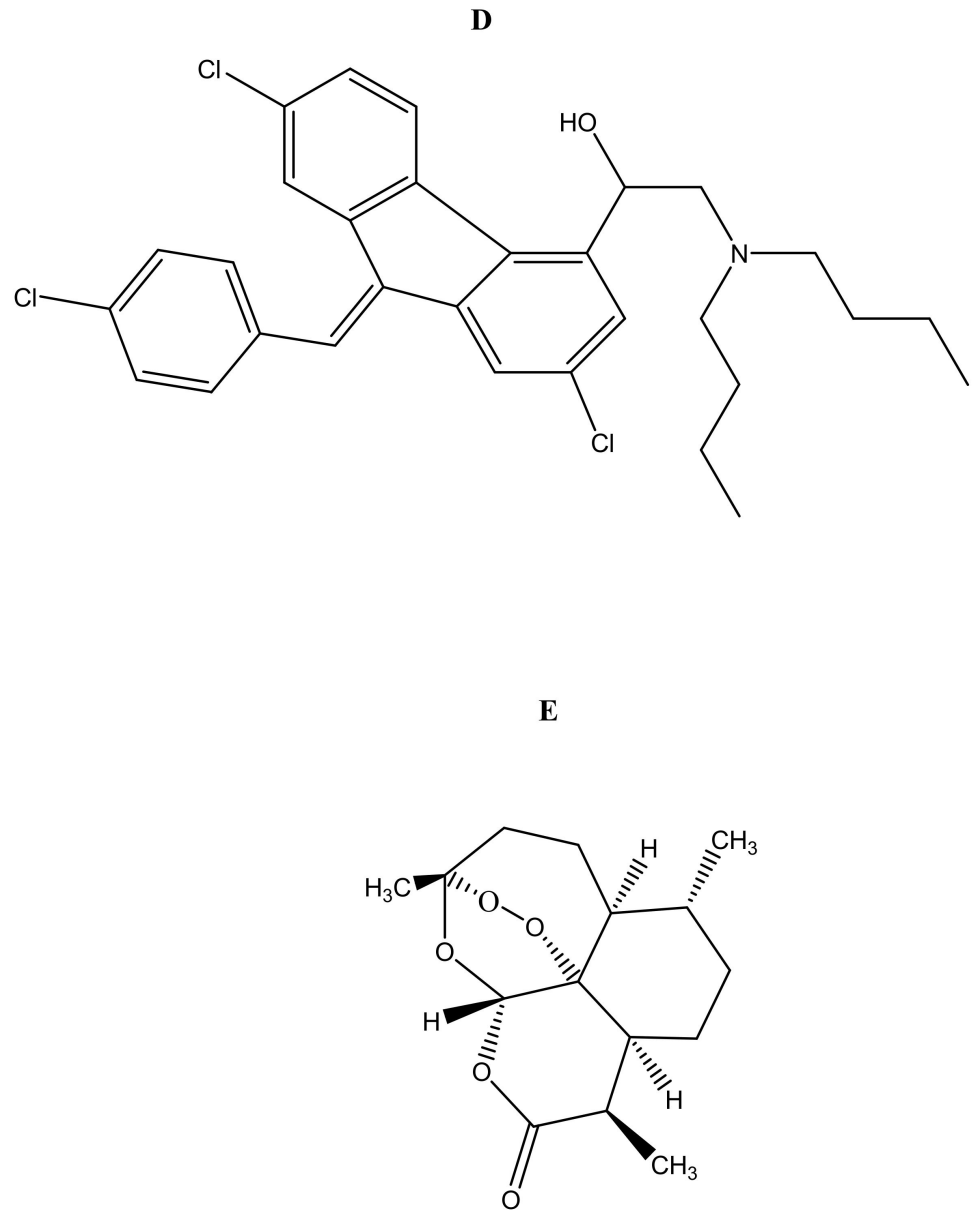
Antimalarials loaded onto polymer based carriers: (**A**) Chloroquine, (**B**) Primaquine, (**C**) Curcumin, (**D**) Lumefantrine, (**E**) Artemisinin.

Malaria is currently treated using antimalarials such as chloroquine (Figure 2a), primaquine (Figure 2b), curcumin (Figure 2c), lumefantrine (Figure 2d) and artemisinin (Figure 2e) and its derivatives. Unfortunately, they all suffer from drug resistance. Drug-resistant malaria is caused by *Plasmodium falciparum*; it causes severe fever and anaemia that leads to more than a million deaths each year [13]. The resistance of antimalarial drugs occurs when malaria parasites survive and multiply in a concentration of a given drug administered that normally kills and prevents their multiplication [14].

There are several factors that influence the emergence and spread of drug-resistant malaria. These factors include the high cost of the treatment, pharmacokinetics mismatch of drugs, incorrect dosage and poor patient compliance [15]. The mechanism of resistance of antimalarials is complex, varies and is influenced by multiple genes [16]. The mechanism of resistance of chloroquine is believed to be due to efflux of the parasite that occurs faster than chloroquine. This efflux expels chloroquine before it reaches the levels required for inhibition to free haem [16]. *P. falciparum* chloroquine resistance transporter (PfCRT) also contributes to the resistance of the parasite to chloroquine drug [17]. Pyrimethamine/sulfadoxine resistance is attributed to the mutations of dihydrofolate reductase (DHFR). These mutations decrease the effectiveness of pyrimethamine/sulfadoxine during the treatment of malaria [18].

### 2.2. Combination Therapy

Combination therapy has been reported to be a good approach in fighting the global challenge of drug-resistant *Plasmodium falciparum* malaria [19]. It is effective in terms of overcoming drug resistance and the long wait for the development of new antimalarial drugs [20]. Sulfadoxine/Pyrimethamine has been used in malaria-endemic countries and has replaced chloroquine as a common drug used in the treatment of uncomplicated malaria in African countries [21]. The World Health Organisation (WHO) proposed Artemisinin-based combination therapies (ACTs), including artemether-lumefantrine, artesunate-sulfadoxine-pyrimethamine, artesunate-amodiaquine, artesunate-mefloquine, and dihydroartemisinin-piperaquine [22].

Mefloquine-artesunate cured 98% of patients with highly resistant falciparum malaria in the Thai-Burmese border [23]. Atovaquone-proguanil was 100% effective in Thai in adults. Chloroquine resistance can be treated with mefloquine or primaquine. Combining either of the drugs for seven days with quinine, mefloquine or artesunate resulted in effective treatment outcomes, exceeding 94% against falciparum malaria in Thailand [24]. Despite the successful application of combination therapy for the treatment of malaria infections, there are some shortcomings associated with the drug combination. These include high cost, different pharmacokinetic profile, toxic and poor availability [25]. Drug resistance is the major cause of the failure treatment outcome of the global management of this disease. In order to overcome drug resistance, different systems have been designed for drug combination, including hydrogels, nanoliposomes, dendrimers, micelles and polymer-drug conjugates [26].

## 3. Polymer-Based Nanocarriers for Drug Delivery

Polymer-based nanocarriers have been designed for biomedical applications as drug delivery systems etc. [27]. They provide several benefits as drug delivery systems such as good biocompatibility, reduced toxicity, improving the bioavailability of the drugs, reducing patient’s compliance, enhancing drug solubility and overcoming drug toxicity [27]. There are different types of systems that have been designed for the delivery of antimalarials such as hydrogels, micelles, nanoliposomes, dendrimers and polymer-drug conjugates etc.

### 3.1. Hydrogels

**Figure 3:**
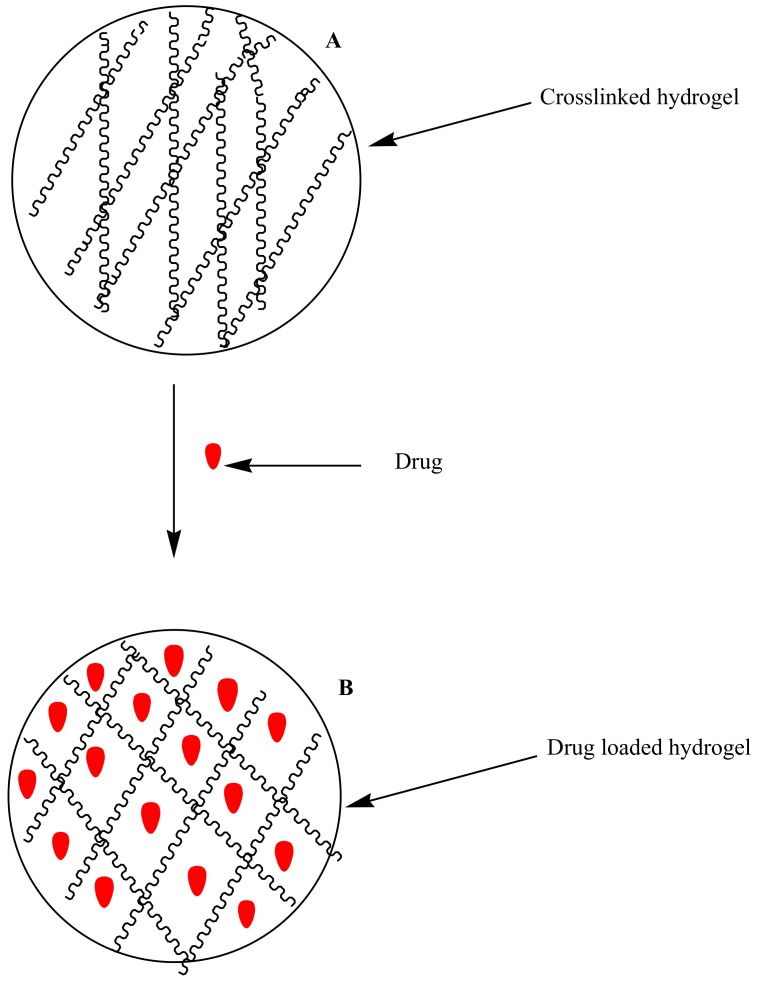
Schematic diagram of: (**A**) cross-linked hydrogel (**B**) Hydrogel encapsulated with drugs.

Hydrogels are three-dimensional polymeric networks prepared from natural and synthetic polymers [28] (Figure 3). They can absorb and retain large quantities of water and biological fluids [28]. Their degree of porosity is influenced by factors such as their method of preparation, the polymer composition and the nature of materials used for their preparation etc. They can be prepared in various forms, such as nanoparticles, slabs, films and microparticles etc. [29]. They exhibit unique properties, such as being affordable, non-toxic, non-immunogenic, biocompatible, environmentally sensitive (e.g, pH, temperature and electric field) and controlling the rate of drug release. They can also be used for combination therapy and are patient compliant. Due to the aforementioned properties, hydrogels are extremely useful in biomedical applications. These include tissue engineering and regenerative medicine, diagnostics, cellular immobilization and the separation of biomolecules etc. [30,31,32]. Some researchers reported hydrogels designed for the delivery of antimalarials.

Aderibigbe et al. prepared hydrogel from gum acacia. The hydrogel was encapsulated with 4-aminoquinoline and curcumin (Table 1). The in vitro results suggested a prolonged and sustained release profile for curcumin, while 4-aminoquinoline exhibited a short-term release profile at 37 °C. The main factor that contributed to the release profile of these drugs was found to be the degree of crosslinking of the hydrogel network. Furthermore, the preliminary studies suggested that hydrogels can be used as dual drug delivery systems for antimalarials with different pharmacokinetics [33]. Dandekar et al. prepared hydrogel nanoparticles from the combination of hydroxyl propyl methyl cellulose and polyvinyl pyrrolidone. The hydrogels were loaded with curcumin. In vivo evaluation of the drug-loaded hydrogels revealed a significant antimalarial activity when compared to curcumin. These results suggest that the formulation is a potential adjunct anti-malarial therapy that can be used along with standard therapy. The formulation was not toxic and it was found to be suitable for a prolonged duration [34]. Aderibigbe and Mhlwatika prepared soy protein isolates–carbopol–polyacrylamide-based gels encapsulated with chloroquine diphosphate and curcumin (Table 1). The hydrogels were pH-sensitive. The in vitro drug release of both drugs from the hydrogels was evaluated. The release mechanism of curcumin was slow and sustained when compared to chloroquine. These findings suggested that the hydrogels can be used for the delivery of two or more antimalarial drugs [35]. Dreve et al. prepared chitosan-based hydrogels loaded with quinine [36]. The concentration of quinine in the hydrogelwas 0.08 mmol and quinine formed chelates in the hydrogels, which were temporary [36]. Liu et al. developed a tough hydrogel that can prolong gastric residence of the drug resulting from the mechanical integrity of the hydrogel, which can withstand the forces which influence gastrointestinal motility. The hydrogel was loaded with lumefantrine and in vivo studies revealed that the relative constant blood drug concentration was maintained over a period of four days after a single administration of the drug loaded-hydrogel. This suggested its potential application for multi-day dosing. Administration of the free lumefantrine resulted in a rapid drug clearance from the blood with a rapid terminal elimination phase. The drug release profile from the hydrogel was a first-order. The rate constant for drug release from the hydrogel in vivo was 0.68 day^−1^, which is threefold higher than the *in vitro* release rate constant. The elimination rate constants for the free drug was 1.17 day^−1^ and that of the formulation was 0.68 day^−1^ indicating a delay in drug elimination from the formulation [37]. Musabayana et al. designed an amidated pectin matrix hydrogel patch for transdermal delivery of chloroquine, in order to mask the bitter taste [38]. In vivo studies on Sprague-Dawley rats indicated that the application of the drug-loaded patch significantly increased Na^+^ excretion compared to the rat in which the drug was administered intravenously in the post-treatment period in animals. This indicated a continued release of residual chloroquine. The results indicate that the transdermal delivery of chloroquine using hydrogel can improve patient compliance [38]. Mavondo et al. developed an asiatic acid loaded and chloroquine-pectin based hydrogel patch for the treatment of malaria [39]. The drug loaded-pectin patch administration in the rats preserved food, water intake and %weight gain when compared to the chloroquine loaded patch, which was decreased. The formulation suppressed parasitaemia significantly. The 5 mg/kg patch of Asiatic acid was the most effective formulation. However, the chloroquine loaded patch exhibited a prolonged time course to clear parasitaemia [39]. Parasitaemia suppression continued until parasites were non-detectable at day 18 for the AA-loaded patch administered animals when compared to the chloroquine-loaded patch, which persisted [39].

### 3.2. Micelles

**Figure 4:**
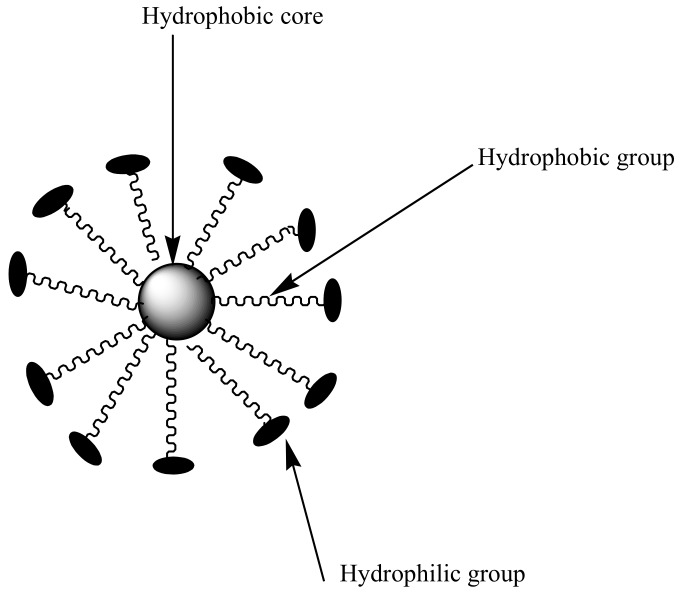
Schematic diagram of micelles.

Polymeric micelles are nanocarriers formed from self-assembling of amphiphilic block copolymers with sizes between 10–200 nm [40] (Figure 4). They are characterized by a hydrophobic core and hydrophilic polymer chains used for the encapsulation of bioactive agents [41]. Hydrophilic chains enhance prolonged circulation time in the blood and provide a sustained drug release mechanism [42]. Micelles with low critical micelle concentration (CMC) are useful for pharmaceutical applications because it contributes to their stability toward dilution in biological fluids. However, micelles with high CMC dissociates, resulting in a dilution effect in the blood [42,43,44]. CMC is influenced by the type of polymer used to prepare the micelles. The only disadvantage with polymeric micelles is that they are difficult to prepare, because their preparation requires a high level of polymer chemistry [45]. The most commonly used hydrophilic chain used in the preparation of micelles is poly (ethylene glycol) (PEG). Others include poly (*N*-vinyl pyrrolidone) (PVP), poly (*N*-isopropyl acrylamide) (pNIPAM) and poly (lactic acid) (PLA) [46].

Manjili et al. prepared methoxypoly (ethylene glycol)-poly (caprolactone)-based micelles [47]. Polyethylene glycol was used as a hydrophilic block and polycapolactone was used as the hydrophobic block. Artemisinin (Figure 2e) was encapsulated within the micelles and the *in vitro* drug release of artemisinin from micelles was sustained. The drug encapsulation efficiency and loading efficiency was 63% and 15%, respectively. AFM results showed that the micelles exhibited a spherical structure [47] (Table 1). A similar formulation was reported by Ramazani et al. [48]. The zeta potential of artemisinin loaded micelles was −8.37 mV, and the average size was 91.87 nm. Artemisinin loading capacity onto the micelles was 19.33% and encapsulation efficacy was 87.21%. *In vivo* antiplasmodial studies indicated that the artemisinin loaded micelles prolonged the drug circulation time and increased the therapeutic efficacy of the loaded drug. These results indicate that artemisinin loaded onto micelles can improve the drug delivery of artemisinin and also be a potential carrier for the treatment of malaria [48] (Table 1). Bhadra et al. developed a formulation of methoxy polyethylene (mPEG) glycol-based micelles loaded with artemether for the controlled release of antimalarial drugs (Table 1). The drug-loaded formulation enhanced the solubility of the loaded drug and also exhibited a sustained release of artemether over a period of 48 h. The CMC of the formulations was determined, and the micelles were stable at a concentration range of 10–30 μg/mL with low CMCs, which was influenced by the generation and type of mPEG used. However, as a result of the slow rate of degradation and hyperbranched micellar, the formulation was found to be toxic [49]. These findings suggest that the design of the micelles influence their therapeutic efficacy [49]. Mannjili et al. synthesized poly (ε-caprolactone)–poly (ethylene glycol)–poly (ε-caprolactone)-based micelles loaded with artemisinin by a nanoprecipitation method [50]. The results average size of the micelles was 83.22 nm and the drug encapsulation efficacy was 89%. In vivo results showed a significantly increased and prolonged drug release when compared to the free drug [50].

### 3.3. Nanoliposomes

**Figure 5:**
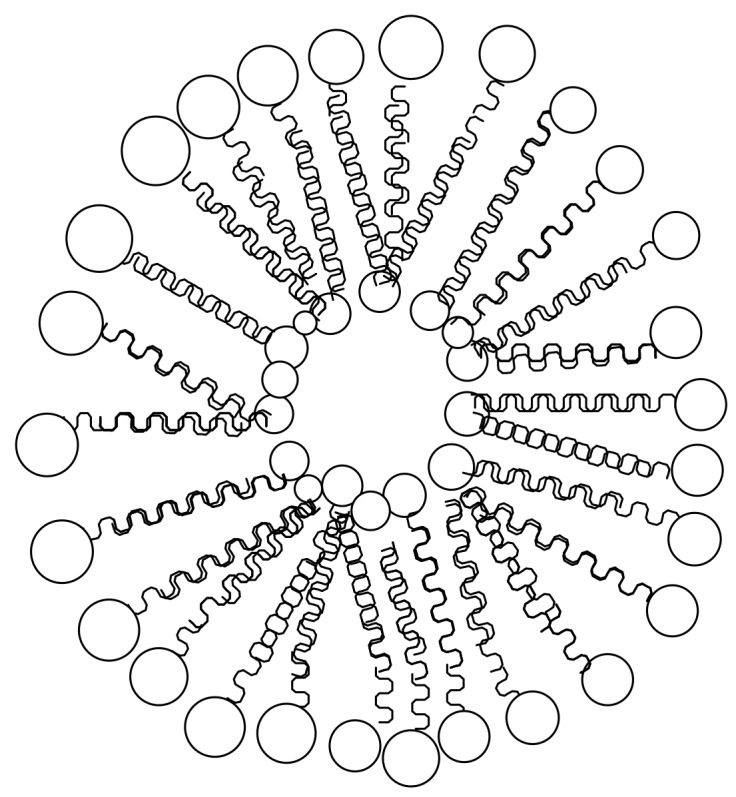
Schematic diagram of nanoliposomes.

Nanoliposomes are molecules composed of a spherical vesicle with an aqueous core surrounded by a lipid bilayer [51] (Figure 5). They are often used for the encapsulation and delivery of bioactive compounds [51]. They are great candidates for drug delivery because of their good biocompatibility and biodegradability. Due to their nanosize, they are able to deliver drugs to the target site [52]. Nanoliposomes are able to enhance the therapeutic efficacy of drugs by modifying drug adsorption, reduce toxicity and metabolism and prolong the drug release mechanism [53]. The main challenges associated with liposomes are that they are expensive to prepare, and there is a possibility of a high rate of drug release during degradation resulting in drug toxicity [54]. Various approaches are used to prepare liposomes in order to overcome the aforementioned limitations.

Chimanuka et al. developed a formulation of liposomes based on egg phosphatidylcholine-cholesterol loaded with an antimalarial drug, beta-artemether (Table 1). The liposomes containing beta-artemether showed good stability over a period of three months on storage at 4 °C without the leakage of beta-artemether. The formulation was further used to treat mice infected with *Plasmodium Chabaudi*. The *in vivo* results showed a 100% cure by clearing the recrudescent parasitaemia [55]. Shakeel et al. combined artemether and lumefantrine onto nanoliposomes for the treatment of malaria (Table 1). The prepared liposomes exhibited drug entrapment efficiencies of 66 and 53.5% for artemether and lumefantrine, respectively. The liposomes were stable at 4 °C for two months without any significant change in the particle size and drug encapsulation efficiencies. The *in vitro* drug release was fast and sustained for a period of 30 h. In vivo toxicity study of the nanoliposomes showed a reduced haemolytic potential. Hepato- and nephrotoxicity analysis showed no sign of fibrosis, fatty infiltration and lymphocyte infiltration confirming the biocompatibility of the nanoliposomes. Based on the results, the prepared nanoliposomes are suitable for treatment of malaria [56]. Marques et al. formulated heparin-based liposomes loaded with primaquine, where heparin acts as antimalarial and targeting moiety (Table 1). The formulation was found to increase antiplasmodial activity in *Plasmodium falciparum* culture by three fold when compared to the free drugs [57]. The antimalarial effect suggested that the formulations interact randomly with Plasmodium-infected red blood cells, which resulted in the lipids entering the cell and reaching the pathogen [57].

Urban et al. developed immunoliposome formulations containing chloroquine diphosphate for the treatment of malaria. *In vitro* results showed that chloroquine delivered from the immunoliposomes exhibited better efficacy than the free drug. Same formulations were prepared and contained chloroquine and fosmidomycin. The liposomes containing chloroquine and fosmidomycin were observed to have a ten-fold increase in malaria activity when compared to the free drugs [58] (Table 1). Rajendran et al. developed liposome-based drug delivery systems prepared from soya phosphatidylcholine, cholesterol-containing either stearylamine or phosphatidic acid with different densities of distearoyl phosphatidylethanolamine-methoxy-polyethylene glycol 2000 [59]. It was loaded with monensin and the formulations were found to be more effective when compared to free monensin in *Plasmodium falciparum* (3D7) cultures and mice models of *Plasmodium berghei* strain NK65 and ANKA. The antimalarial activity of the formulations was influenced by the lipid composition and the formulation preferential uptake by infected red blood cells was significant and prevented parasite recrudescence [59].

### 3.4. Dendrimers

**Figure 6:**
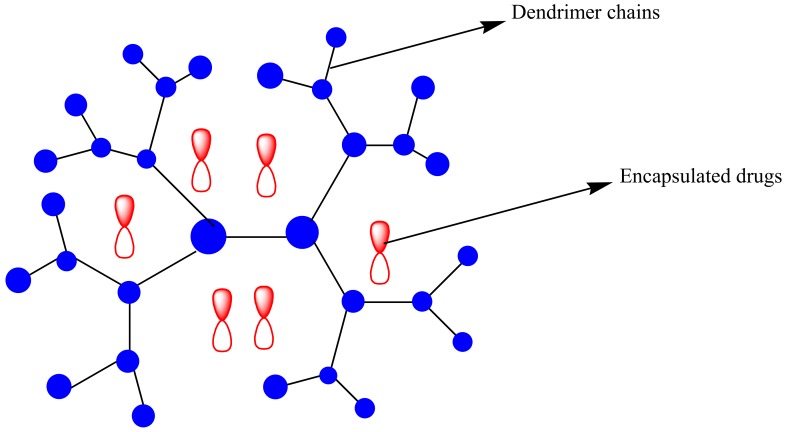
Schematic diagram of dendrimers encapsulated with drugs.

Dendrimers are polymeric materials that are characterized by three dimensional, highly branched and mono-disperse macromolecules [60] (Figure 6). Dendrimers are useful for drug delivery because they are biocompatible; possess low polydispersity index, and precise molecular weight [61]. The exterior layers of dendrimers are made up of functional groups which are very useful for bio-conjugation of drugs and targeting moieties [62]. The interior layers have voids suitable for the encapsulation of drugs with enhanced drug efficacy, reduce drug toxicity and control drug release mechanisms [62]. 

Bhadra et al. formulated polypropylenimine (PPI) dendrimers coated with galactose for the delivery of primaquine phosphate to the liver cells (Table 1). The formulation was prepared by Michael addition and hydrogenation reactions. The in vivo results showed that the coated PPI dendrimers with galactose increased the drug entrapment efficiency. In vitro drug release was sustained over a period of 5–6 days. Based on the results obtained from haemolytic toxicity, blood level and haematological studies suggest that the formulation is safe and suitable for sustained delivery of primaquine to the liver cells [63].

Agrawal et al. synthesized a fourth generation of coated and uncoated poly-l-lysine dendrimers using polyethylene glycol (PEG-1000) as a core for delivery of chloroquine phosphate (Table 1). The formulation of these dendrimers was achieved by protection and deprotection steps of L-lysine via di-BOC (di-tertiary butyl pyrocarbonate). The dendrimers exhibited a controlled drug release mechanism. The coated drug dendrimers exhibited reduced haemolytic toxicity when compared to the uncoated drug loaded dendrimers and the free drug. Ex vivo cellular uptake results further revealed that the uncoated and coated dendrimers exhibited a five times reduction of phagocytosis in macrophages. Furthermore, the haematological results suggested that the coated dendrimers were less immunogenic than the uncoated dendrimers [64].

Movellan et al. prepared amphiphilic dendrimers derived from 2,2-bis (hydroxymethyl) propionic acid (bis-MPA) and pluronic polymers. The dendrimers were encapsulated with antimalarial drugs, such as chloroquine and primaquine for the treatment of malaria (Table 1). The derived dendrimers containing antimalarials were tested in *vitro* against *Plasmodium falciparum* and *in vivo* against *Plasmodium yoelii*. The obtained results suggested that the dendrimers exhibited antimalarial activity with a reduced IC_50_ for chloroquine by 3 fold to 4.0 nm and for primaquine by a 4 fold to 1.1 μm. The dendrimers exhibited a specific targeting to pRBCs when compared to non-infected RBCs [65].

### 3.5. Polymer-Drug Conjugates

**Figure 7:**
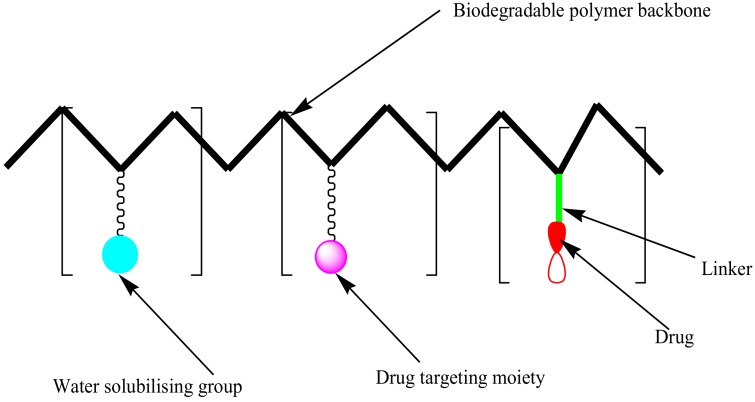
Schematic diagram of polymer-drug conjugates.

Polymer-drug conjugates are systems designed for the incorporation of bioactive agents into polymers via selected functional groups. These include carboxylic acids, alcohols and amino groups [66] (Figure 7). They can be synthesized from linear and branched polymeric carriers. Linear polymers include: poly (vinyl pyrrolidone), polyaspartamides, (PVP) poly (vinyl alcohol), poly (amidoamine), poly (malic acid) and poly (ethylene glycol) (PEG) polymers. Branched polymers: poly (ethyleneimine) dendrimers, poly (amidoamine) (PAMAM) and polymeric micelles [66]. Polymer-drug conjugates exhibit outstanding advantages such as: (1) enhancing the bioavailability of the drugs; (2) improving water solubility and stability of the drugs; (3) non-toxicity and biocompatibility and (4) they are able to deliver the drug to the targeted cells, tissue or organs [67,68]. The concept of polymer-drug conjugates was first reported by Helmut Ringsdorf in 1975 [69]. A polymer-drug conjugate is made up of a biodegradable polymer backbone with three unique units: (1) a hydrophilic unit which makes the whole macromolecules soluble and non-toxic; (2) A drug, which is bound to the polymeric backbone via selected linker; (3) a targeting moiety that transports the system to the desired site [70,71]. The challenges associated with polymer-drug conjugates in combination therapy are the identification of drug ratios and combination, and poor drug loading capacity [72]. Polymer-drug conjugates have been employed for the delivery of antimalarials by several researchers.

Mukaya et al. synthesized polyaspartamide based- conjugates containing platinum (II) complex and bisphosphonate. The preliminary studies of the conjugates and co-conjugates containing bisphosphonate and platinum complex demonstrated an enhanced antimalarial activity when compared to the free chloroquine drug [73]. The Kumar et al. design polymer-drug conjugates encapsulated with primaquine and dihydroartemisinin conjugated onto substituted polyphosphazenes carriers (Table 1). The conjugates were evaluated for their antimalarial efficacy against *Plasmodium berghei* using infected swiss albino mice at different doses. The conjugates exhibited promising antimalarial efficacy at reduced doses when compared to the free drugs [74].

Aderibigbe et al. designed polyamidoamine conjugates from water-soluble polysuccinimide carriers incorporated with aminoquinolines. The prepared conjugates were tested for their in vitro antiplasmodial activity against a chloroquine-sensitive strain of *Plasmodium falciparum*. From the *in vitro* results, it was evident that the conjugates containing 3-diethylamino-1-propylamine solubilizing units were the most active against the chloroquine-sensitive strain of *P. falciparum* [75,76]. Rajic et al. synthesized polyaspartamide conjugates for the delivery of primaquine and glucosamine to the blood cells (Table 1). The conjugates were tested for their antimalarial activity against *Plasmodium berghei* infection in Swiss mice. Conjugates containing primaquine promoted better antimalarial activity than the glucosamine conjugate [77].

## 4. Conclusions

In this article, we explore the various types of polymer-based nanocarriers developed as drug vehicles for the treatment of malaria. The use of polymer-based drug delivery systems provides great benefits in improving the therapeutic efficacy of antimalarials that are currently being used. These benefits include enhancing their water solubility, reducing toxicity, improving drug bioavailability and overcoming drug resistance. Despite the aforementioned advantages, there are some disadvantages associated with some of these systems. These include low drug loading, high cost, toxicity influenced by the preparation approach etc. There is also a pressing need to fully understand the mechanism of action of these systems. Currently, most of the research that has been reported is laboratory work and there is still a need to develop for these systems to reach clinical trial. Based on *in vivo* and in vitro analysis obtained from various researchers, it is clear that polymeric nanocarriers utilized in drug delivery systems are promising candidates that can enhance the therapeutic efficacy of antimalarials, reduce toxicity and overcome drug resistance.

## Figures and Tables

**Table 1:** Classification of antimalarials and their mode of action.

Antimalarials	Classification	Mode of Action	Polymer Carriers	References
Primaquine	Hypnozoiticidal and gametocytocidal	Primaquine interferes with the electron transport in the parasite during respiration process	Nanoliposomes Dendrimers Polymer drug conjugates	57 63, 65 74,77
Chloroquine	Blood schizontocides	Chloroquine accumulate in the acidic food vacuoles of intraerythrocytic trophozoites and thereby prevent haemoglobin degradation	Nanoliposomes Hydrogels Dendrimers	58 38, 39 54, 65
Artemisinin Dihydroartemisinin	Gametocytocidal	Involves the heme-mediated decomposition of the peroxide bridge to produce carbon-centred free radicals	Micelles Polymer-drug conjugates	47, 49 74
Curcumin	Blood schizontocides	Curcumin inhibits the activity of enzymes and lipid peroxides	Hydrogels	33, 34, 35
Artemether Beta-Artemether	Gametocytodal	It acts against erythrocytic stages of *P. falciparum* and inhibits nucleic acid and protein synthesis.	Micelles Nanoliposomes	50 55
Lumefantrine	Blood schizontocides	Lumefantrine is believed inhibits nucleic and formation of β-hematin by forming a complex with hemin	Nanoliposomes Hydrogels	56 37
